# Versorgernetzwerke für Menschen mit Seltenen Erkrankungen: Daten und Expertise bündeln

**DOI:** 10.1007/s00103-022-03592-1

**Published:** 2022-09-27

**Authors:** Holm Graessner, Holger Storf, Franz Schaefer

**Affiliations:** 1 Zentrum für Seltene Erkrankungen (ZSE) Tübingen, Institut für Medizinische Genetik und Angewandte Genomik, Universitätsklinikum Tübingen, Calwerstr. 7, 72076 Tübingen, Deutschland; 2 Institut für Medizininformatik, Universitätsklinikum Frankfurt am Main, Frankfurt am Main, Deutschland; 3 Zentrum für Kinder- und Jugendmedizin, Universitätsklinikum Heidelberg, Heidelberg, Deutschland

**Keywords:** Europäische Referenznetzwerke, Diagnostische Kodierung, Virtuelle Fallkonferenzen, Register, Integration, European Reference Networks, Diagnostic coding, Virtual case conferences, Registries, Integration

## Abstract

Als Seltene Erkrankungen (SE) gelten in der Europäischen Union (EU) Krankheiten, von denen nicht mehr als 5 von 10.000 Menschen betroffen sind. Aufgrund ihrer Seltenheit sind klinische Expertise und qualitätsgesicherte Versorgungsstrukturen rar, die Forschung ist hier im Vergleich zu anderen Krankheiten erschwert. Diese Probleme können jedoch mittels nationaler und länderübergreifender SE-Versorgernetzwerke überwunden werden. Daten und Expertise werden darin gebündelt.

In der Europäischen Union arbeiten die Europäischen Referenznetzwerke (ERN) für Seltene und Komplexe Erkrankungen grenzübergreifend zusammen. Wichtige Leistungen der ERN unter Nutzung von Gesundheitsdaten umfassen die diagnostische Kodierung der SE, die Durchführung von virtuellen, grenzübergreifenden Fallkonferenzen und die Etablierung von europäischen Registern, die zur Messung und Verbesserung der Versorgungsqualität genutzt werden. In den ERN verbinden sich die lokale Datenerzeugung und Dokumentation mit netzwerkweiten Dateninfrastrukturen. In diesem Beitrag werden die datenbasierten Leistungen in und für SE-Versorgernetzwerke beschrieben: 1) diagnostische Kodierung, 2) grenzüberschreitende Fallkonferenzen und 3) ERN-Register für die Versorgung der SE-Patient*innen. Im letzten Abschnitt wird auf die Integration der Netzwerke in die nationalen Gesundheitssysteme eingegangen.

Um einen bestmöglichen Nutzen für die SE-Patient*innen erzielen zu können, müssen die ERN-Aktivitäten und -Strukturen noch besser in die nationalen Gesundheitssysteme integriert werden. In Deutschland nehmen diesbezüglich die Medizininformatik-Initiative und die Deutschen Referenznetzwerke eine zentrale Rolle ein.

## Abstract (en)

In the European Union (EU), rare diseases (RDs) are diseases that affect no more than 5 in 10,000 people. Due to their rarity, clinical expertise and quality-assured care structures are scarce, and research is more difficult compared to other diseases. However, these problems can be overcome by means of national and transnational RD care networks. Data and expertise are pooled in these networks.

In the EU, the European Reference Networks (ERNs) for Rare and Complex Diseases cooperate across borders. Important services provided by ERNs using health data include diagnostic coding of RDs, conducting virtual cross-border case conferences, and establishing European registries that are used to measure and improve the quality of care. In ERNs, local data generation and documentation combine with network-wide data infrastructures. This paper describes the data-based services in and for RD healthcare networks: (1) diagnostic coding, (2) cross-border case conferences, and (3) ERN registries for RD patient care. The final section discusses the integration of the networks into national healthcare systems.

In order to achieve the best possible benefit for SE patients, ERN activities and structures need to be better integrated into national healthcare systems. In Germany, the Medical Informatics Initiative and the German Reference Networks play a central role in this regard.

## Einleitung

In der Europäischen Union werden Krankheiten als „Seltene Erkrankungen“ (SE) bezeichnet, wenn nicht mehr als 5 von 10.000 Menschen betroffen sind. Aufgrund der Seltenheit sind klinische Expertise und qualitätsgesicherte Versorgungsstrukturen rar, die Forschung ist hier im Vergleich zu anderen Krankheiten erschwert. Nationale und länderübergreifende Netzwerke im Bereich der SE sind geeignet diese grundlegenden Probleme zu überwinden. In diesem Artikel wird diese Hypothese anhand der Darstellung und Erläuterung ausgewählter datenbasierter Leistungen der Europäischen Referenznetzwerke (ERN) für Seltene und Komplexe Erkrankungen plausibilisiert. „Seltenheit“ ist dabei konzeptionell und realiter mehrdimensional und bezieht sich auf: die Anzahl der Patient*innen einer SE, die Seltenheit von klinischen Expert*innen für bestimmte SE, die Seltenheit von Wissen und Evidenz über individuelle Erkrankungen und die Seltenheit von qualitätsgesicherten Versorgungsstrukturen.

Netzwerke im Gesundheitswesen stellen eine grundlegende Neuerung für die medizinische Versorgung dar. Für die Leistungserbringung in Versorgernetzwerken ist die Kooperation der Netzwerkteilnehmenden essentiell. Netzwerke entstehen und verstetigen sich nicht von selbst. Als Schlüsselfaktoren für die Entwicklung und den Erfolg von Kooperationsnetzen im Gesundheitswesen wurden die gemeinsame Nutzung von Wissen, ein positives soziales Klima und enge Beziehungen zwischen den Netzwerkpartnern ermittelt. Vor allem die gemeinsame Nutzung von Wissen ist wichtig [1]. Zu den Strategien für die Nutzung von Wissen gehören die Verteilung von Wissen, die Vermittlung von Wissen zur Förderung und Unterstützung der Teilnahme und die Einbeziehung von Interessengruppen in das Netz sowie die Steuerung von Wissen zur Unterstützung der Einrichtung formeller Partnerschaften und Strategien [2]. Die Einbindung der Betroffenen und ihrer Angehörigen in den Wissenstransfer hat sich im Bereich der SE als essentiell erwiesen [3].

ERN für Seltene und Komplexe Erkrankungen adressieren das grundlegende Problem der SE durch grenzübergreifende Zusammenarbeit der wichtigsten Interessensgruppen in diesem Bereich, der Patient*innen und der klinischen Expertisezentren, im europäischen Kontext. Die ERN folgen dabei der Einsicht, dass kein europäischer Staat allein die aus der Seltenheit der SE resultierenden Probleme lösen kann. In Hinsicht auf die Anzahl der Patient*innen und der Expert*innen ist der Mehrwert der Netzwerke offensichtlich. Für die Überwindung der dritten und vierten Dimension der Seltenheit, der Seltenheit von Wissen und Evidenz und der Seltenheit von qualitätsgesicherten Versorgungsstrukturen, ist die Situierung der Netzwerke im digitalen Gesundheitsdatenraum essentiell.

Wichtige Aspekte der Leistungserbringung der ERN basieren auf Datensammlung, Datenaustausch sowie Datenanalyse und Datennutzung. Datenstandards sind dabei ein essentielles Werkzeug zur Ermöglichung dieser Datenaktivitäten (siehe Artikel von Robinson und Graessner in diesem Themenheft [4]). Die diesbezügliche Leistung der ERN umfasst die Ermöglichung und Umsetzung der diagnostischen Kodierung der SE in der Europäischen Union (EU) – um die SE überhaupt in den Gesundheitssystemen sichtbar zu machen –, die Durchführung von virtuellen, grenzübergreifenden Fallkonferenzen und die Etablierung von europäischen Registern, die Versorgungsdaten sammeln und in Hinsicht auf Versorgungsqualität analysieren können.

Allerdings ist für die digitale Leistungserbringung der ERN und die Nutzung der Potenziale des Gesundheitsdatenraumes die Zweitnutzung von Daten wie Laboranalysen, Bildern, klinischen Befunden oder patientengenerierten Befunden notwendig. Im Datenraum der ERN verbinden sich insofern die lokale Datenerzeugung und Dokumentation in den Expertisezentren auf der einen Seite und die ERN-Dateninfrastrukturen auf der anderen Seite. Da die Gesundheitsversorgung in der EU in der Verantwortung der Mitgliedsstaaten liegt, kommt den nationalen Gesundheitssystemen eine integrative Funktion zu. Allerdings wird diese – insbesondere datenintegrative – Funktion gegenwärtig noch nicht bzw. auf sehr heterogene Art und Weise wahrgenommen. In Deutschland werden die Erschließung und Bereitstellung von im klinischen Kontext erfassten Daten für Forschungs- und Versorgungsfragen insbesondere durch die vom Bundesministerium für Bildung und Forschung geförderte Medizininformatik-Initiative (MI-I) adressiert. Im Rahmen dieser entstehen an allen deutschen Universitätskliniken spezialisierte Datenintegrationszentren.

Eine funktionierende Integration der ERN und der nationalen Gesundheitssysteme ist eine essentielle Bedingung für die nachhaltige Realisierung der Potenziale der ERN. Der Aspekt der Integration wird im letzten Abschnitt dieses Artikels im Hinblick auf den Mehrwert datenbasierter Leistungen in und für europäische und deutsche Netzwerke aufgegriffen. Datenbasierte Leistungen sind an dieser Stelle Leistungen, die auf der Basis von standardisiert gesammelten und in digitaler Form vorliegenden Daten erbracht werden. Im nächsten Abschnitt wird zunächst der klinische Mehrwert der genannten 3 datenbasierten Leistungserbringungen, d. h. diagnostische Kodierung, grenzüberschreitende Fallkonferenzen und ERN-Register für die Versorgung der SE-Patient*innen analysiert. Aspekte des Datenschutzes und der Daten-Governance stehen hingegen nicht im Fokus.

## Datenbasierte Leistungen in und für SE-Versorgernetzwerke

### Expertise finden: die Rolle der diagnostischen Kodierung

Durch die im Jahr 2017 vollzogene Etablierung der 24 ERN für jeweils eine SE-Erkrankungsgruppe haben sich Mediziner*innen mit entsprechender Expertise zusammengefunden. Zunächst waren dies über 900 hochspezialisierte Abteilungen aus mehr als 300 Krankenhäusern in 28 Mitgliedsstaaten der EU [5]. Die ERN bilden somit eine gute Anlaufstelle für Personen, die auf der Suche nach entsprechender Expertise sind – dies können behandelnde Ärzt*innen, Betroffene selbst oder deren Angehörige sein. Die teilnehmenden Einrichtungen findet man auf den jeweiligen Webseiten der ERN und der Europäischen Kommission. Eine Übersicht über die deutschen teilnehmenden Einrichtungen gibt der SE-ATLAS (www.se-atlas.de; [6]).

Aufgrund der Designation der ERN-Mitglieder als nationale Expertisezentren, die auch als Schnittstelle zu den nationalen Versorgungsstrukturen fungieren, ist davon auszugehen, dass nicht alle spezialisierten Einrichtungen gleichzeitig auch Mitglied in einem ERN sind. Des Weiteren ist die Spezifikation der Krankheitsexpertise der ERN-Mitglieder auf feingranularer Ebene nur schwer möglich, beispielsweise findet man in der Orphanet-Klassifikation für SE über 2000 Einträge für seltene neurologische Erkrankungen.

Eine große Hoffnung für die Identifikation von Expert*innen für spezifische SE liegt in der Analyse der diagnostischen Kodierung der SE im Behandlungskontext. Die für die Kodierung der Erkrankungen in Deutschland verwendete Internationale statistische Klassifikation der Krankheiten und verwandter Gesundheitsprobleme, 10. Revision, German Modification(ICD-10-GM)-Klassifikation deckt nur einen sehr kleinen Teil der SE direkt ab, bspw. Zystische Fibrose (E84.-) oder das Marfan-Syndrom (Q87.4). Im internationalen Kontext kommt erschwerend hinzu, dass in Deutschland von der Originalversion der Weltgesundheitsorganisation (ICD-10-WHO) abgewichen wird und somit keine einheitliche Basis für eine Recherche zugrunde gelegt werden kann. Hinzu kommt der Einbezug von abrechnungsrelevanten Aspekten bei der Kodierung, welche die Qualität der Datenbasis beeinflussen können.

Eine positive Entwicklung zur Verbesserung der Situation in Deutschland ist, dass dieser Problematik mit der verpflichtenden Kodierung der SE im Rahmen des Digitale-Versorgung-und-Pflege-Modernisierungs-Gesetzes (DVPMG) ab 2023 entgegengewirkt wird [7]. Die Grundlage für die SE-Kodierung bietet die Alpha-ID-SE^1^, welche die Brücke zwischen ICD-10-GM und der Orphanet-Klassifikation herstellt [8]. Auf Basis der im Rahmen der MI‑I geschaffenen technischen und organisatorischen Strukturen ist eine Auswertung der SE-Fallzahlen unter Berücksichtigung des Einverständnisses der Einrichtungen und datenschutzrechtlicher Aspekte perspektivisch denkbar.

In Deutschland sind mittelfristig eine solche Auswertung und Integration im SE-ATLAS geplant. Für eine realistische relative Einordnung der Fallzahlen ist zum einen eine möglichst gute Kodierung der SE und zum anderen ein möglichst vollständiger Zugriff auf die Fallzahlen notwendig. Die Ergebnisse werden entsprechend in die Darstellung der Versorgungseinrichtungen einfließen.

Für die Ermöglichung und Implementierung einer entsprechenden europäischen Versorgungsleistung spielen die ERN eine doppelte Rolle. Erstens stellen sie die Expert*innenbasis bereit, um die ontologische Datenbasis für eine vollständige und granulär adäquate SE-Kodierung zu erarbeiten [9]. An diesem Thema wird auch das im Jahr 2022 beginnende und von Orphanet koordinierte Projekt „Orphanet Data for Rare Diseases“ arbeiten. Zweitens haben die ERN-Mitglieder für die Implementierung der SE-Kodierung im nationalen Kontext eine pilotierende, ausrollende und verstärkende Funktion. Entsprechende Initiativen haben in mehreren europäischen Ländern wie der Tschechischen Republik und Frankreich begonnen. Auf diese Weise werden in der Zukunft länderübergreifende Analysen möglich, um die passende SE-Expertise in der gesamten EU finden zu können.

### Expertise nutzen: grenzüberschreitende Fallkonferenzen

Das Clinical Patient Management System (CPMS) ist eine einzigartige, EU-weite, grenzüberschreitende, digitale Plattform, auf der klinische Expert*innen schwierige Fälle mit internationalen Expert*innen diskutieren können, um Ratschläge einzuholen oder Wissen zum Nutzen von Patient*innen und Kolleg*innen zu teilen. Datenschutzrechtlich und ethisch entspricht das CPMS dem Stand der Dinge.

Aus der Datenperspektive basiert das CPMS auf einer Zweitnutzung von lokal erhobenen Patientendaten. Klinische Daten, Bilder, Videos, Labordaten und genetische Informationen können im CPMS eingegeben bzw. hochgeladen werden und bilden die Datenbasis für eine Fallkonferenz, an der in der Regel ERN-Expert*innen aus mehreren EU-Ländern teilnehmen. Diese datenbasierte Versorgungsleistung ist insofern eine genuine Netzwerkleistung, die die im Netzwerk verfügbare kollektive Intelligenz nutzt, um Wissenslücken zu füllen bzw. neues Wissen gemeinsam zu erzeugen. Auf die Überlegenheit kollektiver Intelligenz gegenüber einzelnen Expert*innen in Hinsicht auf Diagnosefindung haben aktuelle Studien hingewiesen [10–12]. Das CPMS wäre in der Tat ein sehr gut geeignetes Werkzeug, um im Rahmen der grenzüberschreitenden Patientenversorgung [13] eine Gesundheitsdienstleistung zu etablieren, die sehr vielen Menschen mit SE eine bessere Versorgung ermöglicht. Insgesamt wurden seit der Formierung der ERN über 2300 virtuelle Fallkonferenzen im CPMS durchgeführt.

Der systematischen und kosteneffizienten Verwendung des CPMS stehen allerdings einige Probleme im Weg, die im Kontext der Integration der ERN in die nationalen Gesundheitssysteme gelöst werden müssen:

Als Erstes wäre hier das Problem der mangelnden Sensibilisierung (Awareness) der Öffentlichkeit und der Ärzteschaft für die Existenz der ERN und den Umfang der verfügbaren Versorgungsleistungen zu nennen. Die Kenntnis über die Existenz von nationalen Kompetenzzentren und die Möglichkeit einer spezialisierten Beratung ist natürlicherweise die Bedingung für die Wahrnehmung der CPMS-Netzwerkleistung. In der Ärzteschaft sollte bekannt sein, dass die Nutzung des webbasierten CPMS mit einer besseren Zugänglichkeit von Wissen verbunden ist. Fälle können aus der Ferne geprüft und Expertenmeinungen unabhängig vom Standort (oder Land) des oder der Patient*in und der Fachkraft angeboten werden; seltene oder sehr seltene klinische Überlegungen werden oftmals zum ersten Mal von Expert*innen angestellt; die Vorschläge zur Diagnose oder Differenzialdiagnose führen zu diagnostischen Tests und klinischen Folgeuntersuchungen [14].

Das zweite Problem ist der Mangel an etablierten und incentivierten Überweisungswegen für SE, die das CPMS integrieren. Eine Versorgungskette, in der die lokalen Gesundheitssysteme mit den ERN verbunden sind, erleichtert bzw. ermöglicht den Zugang zu hochspezialisierter Gesundheitsversorgung, dem CPMS [15]. Die Zusammenarbeit zwischen den ERN und den nationalen Gesundheitssystemen muss insofern auf lokaler Ebene geklärt und bekannt sein, mit klar definierten Regeln für das Management der Patientenversorgung, einschließlich der Weiterbehandlung von Patient*innen, deren Fall oder Behandlung im CPMS der ERN diskutiert wurden.

Das dritte und letzte hier zu nennende Problem ist die Vergütung der CPMS-Netzwerkleistung. Für alle Patient*innen in Europa, die in einem anderen EU-Staat Gesundheitsleistungen in Anspruch nehmen wollen, sind ihre Rechte klar geregelt. Leistungen werden entsprechend der Erstattung im Heimatland von den Krankenkassen übernommen. Weitaus schwieriger ist die Zuordnung der Leistungserbringung im Fall des CPMS. Die CPMS-Leistungserbringung kann als gemeinsame Wissensnutzung und Wissensbildung genuin nur dem ERN zugeordnet werden. Für eine derartige ERN-Leistung gibt es aber im Augenblick keine Vergütungsmodelle. Komplexitätsreduzierend und problemlösend könnte zukünftig die Bildung der „Europäischen Gesundheitsunion“ [16] wirken, die die strukturierte Beauftragung von transnationalen Gesundheitsdienstleistungen wie den CPMS-Fallkonferenzen ermöglichen könnte.

### Expertise erzeugen und messen: ERN-Register

Die transnationale Vernetzung und Zusammenarbeit einer großen Zahl von spezialisierten klinischen Zentren in den ERN eröffnet die Möglichkeit, Gesundheitsdaten größerer Kohorten von Patient*innen mit SE prospektiv zu sammeln und zur Beantwortung epidemiologischer Fragestellungen, zum Monitoring von Behandlungsqualität und -erfolg sowie für klinische Forschungsinitiativen zu nutzen. Seitdem die ERN kürzlich erweitert wurde, werden in den fast 1600 Referenzzentren der 24 ERN ca. 2 Mio. der insgesamt 30 Mio. EU-Bürger mit SE betreut. Die EU-Kommission fördert die Etablierung von 24 ERN-weiten Registern, in denen mittelfristig alle in den ERN-Zentren betreuten Patient*innen erfasst werden sollen.

Die Organisationsformen der ERN-Register unterscheiden sich in Abhängigkeit von den Vorerfahrungen und Präferenzen der beteiligten Akteure. Während die Mehrzahl der ERN die Dateneingabe in eine zentralisierte Datenbank bevorzugt, organisieren sich einige als „föderierte“ Register ohne Datentransfer und andere durch „orchestrierte“ Integration bereits bestehender krankheitsspezifischer Register. Um den Nutzen der Datensammlung für die Allgemeinheit zu maximieren, sind alle Register den „FAIR“-Prinzipien (Auffindbarkeit, Zugänglichkeit, Interoperabilität, Wiederverwendbarkeit) verpflichtet. Hierfür ist in den Datenmodellen aller Register mit den 16 sogenannten Common Data Elements (CDE) ein einheitlicher Kerndatensatz repräsentiert, der u. a. die klinische und ggf. genetische Diagnose, die Phänotypbeschreibung, Manifestations- und Todesalter sowie eine Erfassung von Morbidität und Lebensqualität enthält. Darüber hinaus erfassen die einzelnen Register in unterschiedlicher Granularität diagnosespezifische Daten zu Behandlung und Krankheitsverlauf [17–19].

Eine noch weitgehend ungelöste Herausforderung stellt die automatisierte Datenerfassung aus den elektronischen Patientenakten (ePA) der Krankenhausinformationssysteme dar. Erste Modellversuche wie das Projekt „Collaboration on Rare Diseases“ (CORD, https://www.medizininformatik-initiative.de/de/CORD) der MI-Initiative in Deutschland sollen zumindest die Extraktion der CDE-Daten ermöglichen, die dann in die ERN-Register übertragen werden könnten. Die Übertragung weiterer krankheitsspezifischer Daten erscheint aber angesichts der noch sehr unvollständig strukturierten Datenerfassung und Fragmentierung der Krankenhausinformationssysteme derzeit noch unrealistisch. Die daher noch unerlässliche manuelle Datensammlung wird von der EU-Kommission finanziell unterstützt.

Die Nutzung der ERN-Registerdaten zur Optimierung der Versorgungsqualität stellt einen wesentlichen Mehrwert der EU-weiten Datensammlung dar. Hierbei definieren die ERN Schlüssel-Indikatoren der Behandlungsqualität (sog. Key Performance Indicators – KPI), die im Rahmen der Register erfasst und zentrumsspezifisch analysiert sowie im Sinne eines Benchmarkings verglichen werden können. Dieses Konzept ist im Register des ERN für Seltene Nierenkrankheiten (ERKReg) bereits realisiert, in dem über 50 KPI kontinuierlich monitoriert werden [17]. Die Nutzer*innen können automatisch aktualisierte Zentrumsstatistiken zu den KPI auf der Register-Homepage aufrufen und die Performance ihres Zentrums mit den registerweiten Durchschnittswerten vergleichen. Die großen im Register erfassten SE-Fallzahlen – in ERKReg wurden innerhalb von 3 Jahren bereits 14.000 Patient*innen registriert – erlauben ein Qualitätsmonitoring, das in keinem Mitgliedstaat isoliert möglich wäre. Die durch die Register ermöglichte Beobachtung der Behandlungserfolge trägt zur europaweiten Harmonisierung und stetigen Verbesserung der Behandlungsqualität bei SE-Patient*innen bei.

Darüber hinaus beabsichtigen die ERN-Register, die gesammelten Daten im Sinne der „Data FAIRness“ [20] der Zweitnutzung zugänglich zu machen. Potenzielle Interessent*innen sind klinische Forscher*innen innerhalb und außerhalb der ERN, nationale und internationale Gesundheitsbehörden, Patientenverbände und andere Nichtregierungsorganisationen sowie die forschende Industrie. Die hierfür jeweils erforderlichen Datenzugangskonzepte werden aktuell von den Data Access Committees der Register erarbeitet. Zur Orientierung über Art und Umfang der verfügbaren Daten wird beim Joint Research Centre (JRC) der EU ein zentrales Metadaten-Verzeichnis angelegt; Abfragen zu den verfügbaren Datensätzen sollen über die „virtuelle Plattform“ des European Joint Programme on Rare Diseases (EJP RD) ermöglicht werden. Langfristig ist die Einbindung der CDE-Datensätze in den Europäischen Gesundheitsdatenraum geplant.

## Integration der ERN in die nationalen Gesundheitssysteme

Die 3 oben beschriebenen datenbasierten Leistungen der ERN, d. h. die diagnostische Kodierung zum Auffinden von Expertise, die grenzüberschreitenden virtuellen Fallkonferenzen und die ERN-Register, erzeugen in Bezug auf die Versorgung der SE-Patient*innen konkreten Mehrwert. Allerdings, und darauf wurde in den unterschiedlichen Kontexten bereits hingewiesen, müssen die ERN-Aktivitäten und -Strukturen in die nationalen Gesundheitssysteme integriert werden, um angepasst auf die Besonderheit der nationalen Systeme einen bestmöglichen Nutzen für die SE-Patient*innen erzielen zu können.

Dieser Logik folgend hat die Europäische Kommission in Abstimmung mit den EU-Mitgliedsstaaten im aktuellen Arbeitsprogramm des EU4Health-Programmes eine sogenannte Coordinated Action ausgeschrieben, in der die nationalen Gesundheitsbehörden zusammenarbeiten sollen, um die Integration der ERN in die nationalen Gesundheitssysteme zu unterstützen [21]. Dabei steht die Gewährleistung der langfristigen Nachhaltigkeit des ERN-Systems im Fokus, die über die Zugänglichkeit des ERN-Systems für SE-Patient*innen und für Angehörige der Gesundheitsberufe auf nationaler, regionaler und lokaler Ebene erreicht werden soll [22]. Eine bessere Integration der ERN in die nationalen Gesundheitssysteme wird darin im Wesentlichen angestrebt über

1. die Ausarbeitung eines Konzepts für eine nationale Verbreitungs- und Kommunikationsstrategie über die ERN,
2. die Entwicklung von Überweisungssystemen an die ERN, einschließlich Leitlinien für die Einbeziehung von CPMS-Ratschlägen in die Patientenversorgung und Empfehlungen zu CPMS-Erstattungsmodellen, und
3. die Etablierung nationaler Qualitätssicherungsmodelle für Seltene und Komplexe Erkrankungen und Empfehlungen für die Organisation klar definierter nationaler Versorgungspfade, die mit ERN in Verbindung stehen.

Diese Maßnahmen sind unmittelbar anschlussfähig an die beschriebenen digitalen ERN-Leistungen. Als wichtige datenbasierte Grundlage wird die Gewährleistung der Interoperabilität zwischen nationalen und lokalen Datenstrukturen und ERN-Datenstrukturen (einschließlich ERN-Register und CPMS) genannt.

Die Umsetzung der 3 aufgeführten Maßnahmen in Kombination mit der Interoperabilität der Datenstrukturen würde auch im Kontext des deutschen Gesundheitssystems die optimale Nutzung der Potenziale der digitalen Leistungserbringung der ERN ermöglichen. Mittels der durch die Medizininformatik-Initiative (MI-I) sukzessive ausgebauten interoperablen Datenstrukturen und durch den speziellen Fokus des „MI‑I SE Use Case CORD“ sind in der Tat gute Voraussetzungen für die Ermittlung und Analyse der SE-Expertise mittels diagnostischer Kodierung, für die Bereitstellung von Daten für grenzüberschreitende virtuelle Fallkonferenzen und für die Datenextraktion und Datenübermittlung in Richtung der ERN-Register geschaffen worden. Für eine gelungene Schnittstelle der deutschen Versorgungsaktivitäten und Versorgungsstrukturen mit den ERN-Pendants, die in 24 SE-Erkrankungsgruppen organisiert sind, bedarf es jedoch auch einer anschlussfähigen Versorgungsorganisation in Deutschland. Tatsächlich nennt die Ausschreibung der Europäischen Kommission mit der „Entwicklung nationaler Netze für seltene Krankheiten“ die angestrebte nationale Versorgungsinnovation. Die entsprechende Bildung „Deutscher Referenznetzwerke“, mit der im Jahr 2020 begonnen wurde [23], wäre insofern nicht nur für die Bündelung und optimale Nutzung der in Deutschland vorhandenen SE-Expertise und für die Schnittstelle zu den ERN zentral, sondern auch für die Transformation der Leistungserbringung in Richtung digitale Gesundheitsversorgung.

## Fazit

Die in 2017 gebildeten Europäischen Referenznetzwerke für Seltene und Komplexe Erkrankungen sind für die Versorgung der Patient*innen mit SE die wichtigste Infrastruktur in der EU. Die Leistungserbringung der ERN basiert wesentlich auf Gesundheitsdaten und umfasst u. a. die Ermöglichung und Umsetzung der diagnostischen Kodierung der SE in der EU, die Durchführung von virtuellen, grenzübergreifenden Fallkonferenzen und die Etablierung von europäischen Registern. Die optimale Nutzung der Potenziale der digitalen Leistungserbringung der ERN für das deutsche Gesundheitssystem wird von der Integration der ERN abhängen. Anschlussfähige und innovative Versorgungsstrukturen sind daher notwendig und mit der Bildung der „Deutschen Referenznetzwerke“ auf den Weg gebracht worden.

## Footnotes

1 Die Alpha-ID ist eine Identifikationsnummer für Diagnosen und ermöglicht, medizinische und alltagssprachliche Bezeichnungen zu kodieren. Eine Alpha-ID ist demnach eine fortlaufende, nicht-sprechende, stabile Identifikationsnummer, die jedem Eintrag des alphabetischen Verzeichnisses zugeordnet ist. Somit kann der Eintrag eindeutig identifiziert werden. Die Alpha-ID-SE ist eine erweiterte Variante der Alpha-ID, die neben dem ICD-10-GM-Code zusätzlich die sogenannte Orpha-Kennnummer für Diagnosenbezeichnungen von Seltenen Erkrankungen enthält.
